# A phantom study investigating the relationship between ground‐glass opacity visibility and physical detectability index in low‐dose chest computed tomography

**DOI:** 10.1120/jacmp.v16i4.5001

**Published:** 2015-07-08

**Authors:** Katsuhiro Ichikawa, Takeshi Kobayashi, Motoyasu Sagawa, Ayako Katagiri, Yukiko Uno, Ryo Nishioka, Jun Matsuyama

**Affiliations:** ^1^ Department of Quantum Medical Technology Faculty of Health Sciences, Institute of Medical, Pharmaceutical and Health Sciences, Kanazawa University Ishikawa; ^2^ Department of Radiology Ishikawa Prefectural Central Hospital Ishikawa Japan; ^3^ Department of Thoracic Surgery Kanazawa Medical University Ishikawa Japan

**Keywords:** computed tomography, ground‐glass opacity, detectability, dose index, lung cancer screening

## Abstract

In this study, the relationship between ground‐glass opacity (GGO) visibility and physical detectability index in low‐dose computed tomography (LDCT) for lung cancer screening was investigated. An anthropomorphic chest phantom that included synthetic GGOs with CT numbers of ‐630 Hounsfield units (HU; high attenuation GGO: HGGO) and ‐800 HU (low attenuation GGO: LGGO), and three phantoms for physical measurements were employed. The phantoms were scanned using 12 CT systems located in 11 screening centers in Japan. The slice thicknesses and CT dose indices (${\text{CTDI}}_{\text{vol}}$) varied over 1.0–5.0 mm and 0.85–3.30 mGy, respectively, and several reconstruction kernels were used. Physical detectability index values were calculated from measurements of resolution, noise, and slice thickness properties for all image sets. Five radiologists and one thoracic surgeon, blind to one another's observations, evaluated GGO visibility using a five‐point scoring system. The physical detectability index correlated reasonably well with the GGO visibility ($R^{2}=0.709,p<0.01$ for 6 mm HGGO and $R^{2}=0.646,p<0.01$ for 10 mm LGGO), and was nearly proportional to the CTDI_vol_. Consequently, the ${\text{CTDI}}_{\text{vol}}$ also correlated reasonably well with the GGO visibility ($R^{2}=0.701,p<0.01$ for 6 mm HGGO and $R^{2}=0.680,p<0.01$ for 10 mm LGGO). As a result, the ${\text{CTDI}}_{\text{vol}}$ was nearly dominant in the GGO visibility for image sets with different reconstruction kernels and slice thicknesses, used in this study.

PACS numbers: 81.70.Tx, 87.57.Q‐

## I. INTRODUCTION

Low‐dose computed tomography (CT) for lung cancer screening has been well established as a more effective method of detecting nodules and lung cancers, including early‐stage cancers, than plain chest radiography.^1^, ^2^, ^3^, ^4^, ^5^, ^6^, ^7^, ^8^ In low‐dose CT for lung cancer screening (LDCT), the detection task is typically the observation of focal lung opacities or nodules. For the task, the selection of scanning parameters is important because image characteristics vary as a function of the chosen scanning parameters.^(9)^ Ground‐glass opacities (GGOs) are more difficult to detect than solid nodules because of their characteristic subtle contrasts.^10^, ^11^ In addition, the reduced radiation dose delivered in LDCT degrades image quality, causing the resultant image characteristics to affect GGO detectability.^(12)^

It is well known that noise in a CT image is affected not only by the dose level but also by the slice thickness and reconstruction kernel.^(13)^ Reduced slice thicknesses and sharper reconstruction kernels result in more image noise in the CT image. In addition, these parameters noticeably affect the sharpness of an object's edge. For these reasons, the effect of acquisition and reconstruction parameters on GGO detectability has been of long‐standing interest in the investigation of LDCT performance. However, most previous reports on the technical aspects of LDCT have only studied the effects of dose level and/or image noise (i.e., standard deviation).^14^, ^15^, ^16^ To our knowledge, only a single published report has described the results of modulation transfer function (MTF) as a resolution property index.^(9)^ The study provided additional insight into LDCT performance analysis, but did not consider either the noise power spectrum (NPS) as a noise property index or the slice sensitivity profile as a measure to quantify image quality.

The purpose of this phantom‐based study was to investigate the relationship between GGO visibility and physical detectability index on the basis of a signal‐to‐noise ratio model in LDCT. To investigate this relationship, we analyzed sets of images over a wide range of imaging protocols that were obtained using 12 multidetector row CT (MDCT) systems.

## II. MATERIALS AND METHODS

### A. Screening centers and CT systems

Eleven screening centers in Japan housing a total of 12 MDCT systems participated in this study. An anthropomorphic chest phantom and three phantoms for physical measurements of spatial resolution, noise, and slice sensitivity profile, described in the later sections, were sent to each center and scanned using specified parameters. Each phantom scan resulted in a total of 13 image sets because one of the MDCT systems reconstructed two image sets with different slice thicknesses. The MDCT systems used in this study each had 2–64 data acquisition channels.

### B. GGO visibility study

#### B.1 Synthetic GGOs and phantom scanning

**Figure 1:**
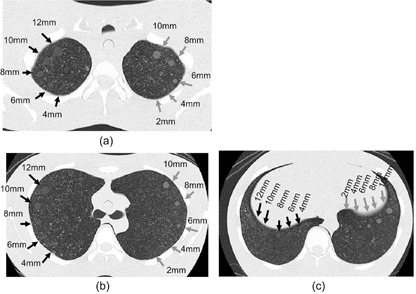
Locations of synthetic nodules at (a) upper, (b) middle, and (c) lower lung fields in the chest phantom. Black and gray arrows indicate synthetic ground‐glass opacities with ‐800 HU (LGGOs) and ground‐glass opacities with ‐630 HU (HGGOs), respectively.

The anthropomorphic chest phantom (LSCT001; Kyoto Kagaku Co., Kyoto, Japan)^12^, ^15^, ^16^, ^17^ used in this study has a chest circumference of 930 mm and a height of 370 mm, modeling Japanese adult men of 40 years of age or older. The materials used in the phantom are a radiographic water‐equivalent substance for the chest wall and mediastinum and a radiographic bone‐equivalent substance for the vertebrae and ribs. The simulated lungs are made of a composite of Styrofoam and hard urethane foam powder in urethane resin adhesive. In the chest phantom, five synthetic GGOs (spherical objects) with CT numbers of ‐630 Hounsfield units (HU) having diameters of 2, 4, 6, 8, and 10 mm (high attenuation GGOs: HGGOs) and five synthetic GGOs with CT numbers of ‐800 HU having diameters of 4, 6, 8, 10, and 12 mm (low attenuation GGOs: LGGOs) were each located in the upper, middle, and lower lung fields, as shown in Fig. 1. The CT values of synthetic GGOs corresponded to a tube voltage of 120 kV. Scan and reconstruction parameters were set to values typically used in the screening protocols for each center. Table 1 shows the number of data acquisition channels, manufacturer name, scan and reconstruction parameters, and volume CT dose index (${\text{CTDI}}_{\text{vol}}$) corresponding to each image set. The nominal slice thickness and ${\text{CTDI}}_{\text{vol}}$ ranged from 1.0 to 5.0 mm and 0.85 to 3.30 mGy, respectively. The reconstruction intervals were set to half of each nominal slice thickness. We used the ${\text{CTDI}}_{\text{vol}}$ values indicated on the operation console of each MDCT system, or the value in the technical information provided by the individual CT manufacturer. Since the indicated ${\text{CTDI}}_{\text{vol}}$ has a tolerance of 20% according to the related International Electrotechnical Commission (IEC) standard,^(18)^ ${\text{CTDI}}_{\text{vol}}$ may actually have to be measured by a method standardized by IEC^(19)^ when a higher accuracy is desired. However, it has been reported that the relative disagreement between nominal and measured ${\text{CTDI}}_{\text{vol}}$ values is under 5%, except in the case of a pediatric abdomen protocol,^(20)^ and that relative errors for repeated weighted CT dose index measurements are also under 5%.^(21)^ On the basis of these results, we decided to use the scanner‐indicated ${\text{CTDI}}_{\text{vol}}$ values. For image sets scanned using automatic exposure control (AEC) functions, the average tube current‐time products of all images in each set were calculated and the corresponding ${\text{CTDI}}_{\text{vol}}$ values were estimated. The estimated effective dose ranged from 0.36 to 1.39 mSv with an assumed scan length of 30 cm and conversion factor of 0.014 mSv/mGy/cm for the chest, as indicated in International Commission on Radiological Protection (ICRP) publication 102.

In Japan, the Japanese Society of CT Screening (JSCTS) has set a guideline of an effective dose reference level of 1.1 mSv, and recommends a slice thickness of 5 mm or less for MDCT systems.^(22)^ However, this JSCTS guideline does not provide any detailed recommendations regarding slice thickness and reconstruction kernel type. Therefore, the nominal slice thicknesses, reconstruction kernels, and ${\text{CTDI}}_{\text{vol}}$ of the image sets were not standardized among the 11 participating screening centers. We took into account these varieties in scanning parameters in our investigation.

**Table 1:** Number of data acquisition channels, manufacturer name, scan and reconstruction parameters, and ${\text{CTDI}}_{\text{vol}}$ value for each image set

*Image Set*	*Data Acquisition Channel*	*Manufacturer*	*Tube Voltage (kV)*	*Tube Current Time Product (mAs)*	*AEC*	*Detector Config*.	*Pitch Factor*	*Slice Thickness (mm)*	*Reconstruction Kernel*	*CTDI_vol_ (mGy)*
*A*	*4*	Hitachi	120	40		2.5 mm×4	1.375	5.0	Lung‐9	2.85
B	*4*	Toshiba	120	22.5		5 mm×4	1.125	5.0	FC50	2.10
C	16	Toshiba	120	22.5		1 mm×16	1.438	3.0	FC52	2.00
D	64	Philips	120	15	On	0.625 mm×4	0.985	2.0	Lung‐enhance	1.90
E	2	GE	120	40		5 mm×2	1.500	5.0	Chest	2.60
F	4	Toshiba	120	24		3 mm×4	1.375	3.0	FC53	2.07
G	4	Toshiba	120	27.75	On	3 mm×4	1.375	3.0	FC53	2.40
H	4	Toshiba	120	33.75	On	3 mm×4	1.875	5.0	FC52	2.13
I	16	Toshiba	120	30		1 mm×16	0.938	1.0	FC51	3.30
J	16	Toshiba	120	30		1 mm×16	0.938	2.0	FC52	3.30
K	4	GE	120	12		2.5 mm×4	0.750	2.5	Standard	1.02
L	4	Hitachi	120	8		5 mm×4	0.750	5.0	Kernel‐20	0.85
M	16	GE	120	42.4	On	1.25 mm×16	1.375	3.75	BonePlus	2.48

#### B.2 Visual evaluation

Image sets of the chest phantom were collected and sent to the second author's hospital (not one of the screening centers) where the visibilities of GGOs were evaluated by a central review team comprising one board‐certified chest radiologist (with 24 yrs of experience), two board‐certified general radiologists (with 10 and 12 yrs of experience), two residents (each with 2 yrs of experience), and one thoracic surgeon (with 22 yrs of experience). Each observer performed the evaluation individually and was blind to evaluations of other members of the review team. A five‐point visual evaluation scoring system was adopted to classify the synthetic GGOs in the image sets as follows: 1) invisible; 2) subtly visible; 3) visible with a slight chance of missing detection; 4) visible; and 5) clearly visible. Prior to evaluation, the observers agreed that a score of 3 is the minimum level to be considered for the detection of a GGO, through a joint training to look at example images. Since the synthetic GGOs were located at three locations (upper, middle, and lower lung field), observers evaluated the visibility score at each location. We excluded the 4 mm LGGOs and the 2 mm HGGOs from the evaluation because they were too subtle to be recognized in most of the image sets.

An image‐viewing station with a 1.3 megapixel 19‐inch color display calibrated at a maximum luminance of 270‐cd/m^2^ and a contrast of 500:1 was used for the visual evaluation. During the observation, a fixed window condition was used (window width, 1500 HU; window center, ‐600 HU). Although the image set order for the observation was not randomized (used order: A–M), the observers had no knowledge of the scan and reconstruction parameters for each image set. These parameters were hidden in the image display.

### C. Physical image evaluation

#### C.1 Detectability index

We used a matched filter signal‐to‐noise (SNR) to evaluate the physical detectability of low‐contrast nodules.^(23)^ This matched filter SNR (${\text{SNR}}_{M}$) can be calculated by

(1) $$SNR_{M}{\;}^{2}=2\pi \int_{0}^{\infty }u\frac{S^{2}(u)MTF^{2}(u)}{NPS(u)}du$$

where *u* is the spatial frequency and S(u) denotes the spatial frequency spectrum of the signal. The MTF(u) and NPS(u) were measured using a wire phantom and a water phantom, respectively,^13^, ^24^, ^25^, ^26^, ^27^ as described in Material & Methods section C.2 and C.3 below. S(u) was calculated using a numerical simulation, as presented in section C.4 below.

#### C.2 MTF measurement

The MTF of each image set was measured by a wire method that has been previously reported.^13^, ^24^, ^25^ Here, a 50 mm diameter cylindrical water phantom containing a 0.15 mm thin copper wire aligned parallel to the phantom axis was used. The phantom was placed in such a way that the wire was precisely aligned perpendicular to the scan plane. The scan and reconstruction parameters were set to the values used for the chest phantom, except for the reconstruction field of view (FOV). The FOV was set to 50 mm (or to the minimum size if the 50 mm setting was not possible) to obtain a correct impulse response with sufficient data points (i.e., a sufficiently small pixel pitch).^(25)^ A subimage with 256×256 pixels centered on the wire was extracted from each wire CT image. The two‐dimensional (2D) Fourier transform of the subimage was then performed. The 2D result was converted to a one‐dimensional (1D) result using an azimuthal averaging. Finally, the result was divided by the magnitude obtained at zero frequency to yield the MTF.

#### C.3 NPS measurement

The NPS of each image set was measured using a cylindrical water phantom with a diameter of 200 mm. Scan and reconstruction parameters were set to the same values as that used for the chest phantom, except for the FOV, which was set to 200 mm to unify the pixel sizes (frequency ranges of the NPS results). Prior to the measurements in the screening centers, we compared the SD values between the water phantom and the chest phantom images to confirm that the water phantom provided a similar noise level (attenuation) to the chest phantom at the tube voltage of 120 kV. For this investigation we used a 16‐channel MDCT system, Somatom Emotion (Siemens Medical Systems, Munich, Germany). The water and chest phantoms were scanned at 120 kV, 100 mAs, and then the CT images were reconstructed with a nominal slice thickness of 2 mm and reconstruction kernel of B31 for standard abdomen. The SD values of the water phantom images were measured in a ROI of 100×100 pixels at the center of image. Five ROIs each with 10×10 pixels were carefully placed in the middle lung field of each image set, so that the ROIs did not contain any observable structures, and then the five SD values were averaged. The SD value of the water phantom was 15.4% higher than that of the chest phantom. Since there was no large attenuation difference between the water and chest phantoms, we decided to use the water phantom for convenience in the NPS measurement. The resultant SD difference measured from the water and chest phantom image sets obtained in the screening centers was 16.3%±6.1%.

The NPS for the water phantom image was calculated using a 2D Fourier transform according to a previously published method.^13^, ^26^, ^27^ To generate a 1D NPS representation, radial rebinning of the data points comprising the 2D NPS into 25 frequency bins, was performed, and these bins were then averaged.

#### C.4 S(u) calculation using simulated slice image of GGO nodule

S(u) was calculated from numerically simulated slice images of spherical objects corresponding to HGGO and LGGO. First, spherical numerical object data with $0.2\times 0.2\times 0.2\;{\text{mm}}^{3}$ voxels with values of 100 for LGGOs and 270 for HGGOs were created. The voxel values were determined by the CT number difference between the lung field (‐900) of the chest phantom and the synthetic nodules (‐800 and ‐630). The diameters of the spherical objects were set to 6 mm for HGGO and 10 mm for LGGO, corresponding to target sizes, as described in the Material & Methods section D and the Results section A. To obtain the simulated slice image from the voxel data, the data points at each x–y location were then summed along the z‐axis using a weighted function corresponding to the slice sensitivity profile (SSP) of each image set.

The SSP of each image set was measured using a lead bead point source^(28)^ of 0.2 mm diameter, which was enclosed in a cylindrical acrylic phantom with a diameter of 50 mm and a length of 100 mm. Scan and reconstruction parameters were set to the values used for the chest phantom, except for the FOV and slice interval. The slice interval was set to one‐tenth of the nominal slice thickness to obtain data plots with sufficiently fine increments. The FOV was set to 100 mm to obtain clear visualization of the bead, which can be used for its intensity measurement. Intensity values were measured across the obtained images. Data were then normalized to the peak value after the subtraction process using the background level. Finally, the SSP profile data were interpolated into the weight function data with a 0.2 mm data interval.

**Figure 2:**
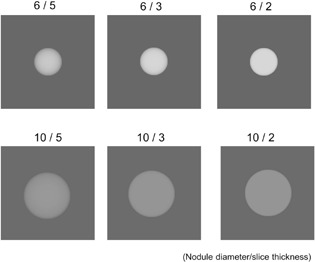
Examples of simulated slice image of spherical objects (nodules) with 6 and 10 mm diameters. Signal spectra used for the physical detectability index were calculated from these images.

Figure 2 shows examples of the generated slice images. The images simulated noiseless sliced images of nodules, and therefore, could be used for the calculation of S(u). Image data were trimmed to 256×256 pixels around the object, analyzed by a 2D fast Fourier transform algorithm, and then S(u) was extracted from the x‐axis data in the calculated 2D spectrum as a 1D spectrum.

### D. Target sizes of HGGO and LGGO

From previous reports,^11^, ^12^, ^29^ HGGOs with a CT number of ‐630 HU were considered to have sufficient contrast to simulate the detection of clinical GGOs. In addition, many screening programs have adopted a 5 mm diameter nodule size as the cutoff between positive and negative nodules on CT.^1^, ^2^, ^3^, ^4^, ^5^, ^6^, ^7^ Therefore, we used 6 mm HGGOs and analyzed the relationship between the visibility, SNR_M_^2^ and CTDI_vol_. From this perspective, there were no criteria available to determine the target size of LGGOs, as the attenuation of LGGO was too low. Thus, we selected a LGGO size that showed a clearly visible difference between image sets and had an adequate median of visibility level, by inspecting distributions of the visibility results for 12, 10, 8, and 6 mm LGGOs, as described below.

## III. RESULTS

### A. Visibility scores for HGGO and LGGO

**Figure 3:**
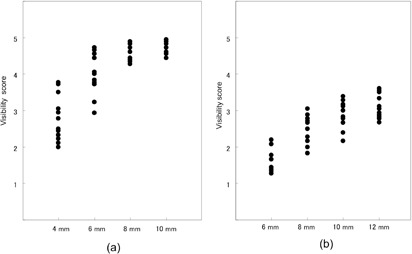
Distributions of averaged visibility scores for respective image sets, as a function of nodule size for (a) HGGO and (b) LGGO.

Figures 3(a) and 3(b) show the distributions of averaged visibility scores of respective image sets, as a function of nodule size for HGGO and LGGO, respectively. A wide distribution was observed in the plots of 8 mm and 10 mm LGGOs; however, the visibility of 8 mm LGGOs (median: 2.31) were thought to be insufficient for the GGO detection. We thus decided to select 10 mm as the target LGGO size for analyzing the relationship between the visibility, SNR_M_^2^ and CTDI_vol_.

### B. MTF

**Figure 4:**
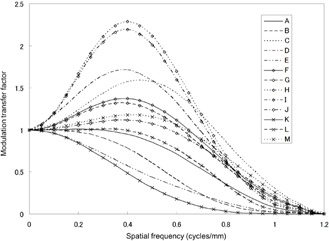
Measured MTFs of image sets. Some of the edge enhancement‐type kernels (image sets of C, D, H, and J) presented noticeably higher MTF values exceeding 1.0 in regions with low and medium frequencies.

Figure 4 shows the measured MTFs for the respective image sets. A wide variety of resolution properties corresponding to the respective kernel types set in the image sets are shown. Some of the edge enhancement‐type kernels (image sets of C, D, H, and J) presented markedly higher MTF values exceeding 1.0 in regions with low and medium frequencies.

### C. Relationship between SNR_M_^2^, GGO visibility, and CTDI_vol_

**Figure 5:**
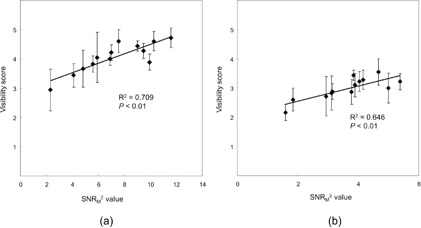
Plots of SNR_M_^2^ vs. average visibility score for (a) 6 mm HGGOs and (b) 10 mm LGGOs. Regression lines are also shown.

Figure 5 shows plots of SNR_M_^2^ versus the average score for 6 mm HGGOs and 10 mm LGGOs. Each plot corresponds to an image set, and the error bar represents the 95% confidence interval calculated across the six observers. Reasonable correlations were observed for HGGO ($R^{2}=0.709,p<0.01$) and LGGO ($R^{2}=0.646,p<0.01$).

**Figure 6:**
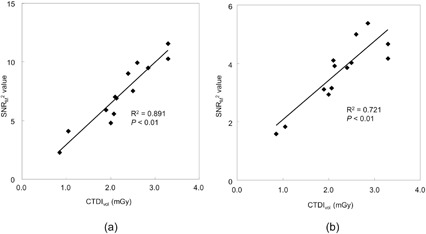
Plots of ${\text{CTDI}}_{\text{vol}}$ vs. SNR_M_^2^ for (a) 6 mm HGGOs and (b) 10 mm LGGOs. Regression lines are also shown.

**Figure 7:**
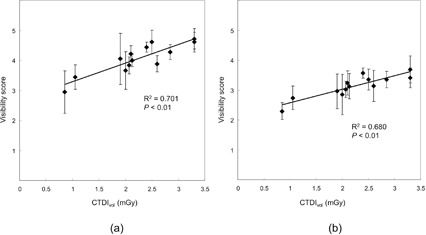
Plots of ${\text{CTDI}}_{\text{vol}}$ vs. average visibility score (a) for 6 mm HGGOs and (b) 10 mm LGGOs. Regression lines are also shown.

Relationships between ${\text{CTDI}}_{\text{vol}}$ and SNR_M_^2^ values for 6 mm HGGOs and 10 mm LGGOs are presented in Fig. 6. SNR_M_^2^ is nearly proportional to ${\text{CTDI}}_{\text{vol}}$ for both HGGO ($R^{2}=0.891,p<0.01$) and LGGO ($R^{2}=0.721,p<0.01$), while less proportionality is observed in the high‐dose region for LGGO. Figure 7 shows the plots of ${\text{CTDI}}_{\text{vol}}$ versus the average score for 6 mm HGGOs and 10 mm LGGOs. Reasonable correlations are also observed for HGGO ($R^{2}=0.701,p<0.01$) and LGGO ($R^{2}=0.701,p<0.01$ for 6 mm HGGO and $R^{2}=0.680,p<0.01$).

Table 2 shows the ranks of the visibility score, SNR_M_^2^, and ${\text{CTDI}}_{\text{vol}}$ for the image sets. The similarity between the three ranked sets was examined using the Kendall rank correlation test. As a result of the test, the rank consistencies between the three sets were demonstrated at p<0.01 for both HGGO and LGGO ($\chi^{2}=31.43>\chi_{0.01}^{2}=26.22$ for HGGO, $\chi^{2}=32.97>\chi_{0.01}^{2}=26.22$ for LGGO).

**Table 2:** Ranks of image sets for score rate, SNR_M_^2^, and CTDI_vol_: 6 mm HGGOs, 10 mm LGGOs

		*Image Set*												
		*A*	*B*	*C*	*D*	*E*	*F*	*G*	*H*	*I*	*J*	*K*	*L*	*M*
6 mm HGGOs	Visibility score	9	8	3	7	5	4	10	6	11	13	2	1	12
	*SNR*_*M*_^2^	10	7	3	5	11	4	9	6	12	13	2	1	8
	CTDI_vol_	11	7	4	3	10	5	8	6	12	13	2	1	9
10 mm LGGOs	Visibility scores	9	8	3	4	7	5	12	6	11	13	2	1	10
	*SN*R_*M*_^2^	13	10	3	4	13	5	6	9	8	11	2	1	7
	CTDI_vol_	11	6	4	3	10	5	8	7	12	13	2	1	9

### D. Image examples

Close‐up image examples of HGGOs and LGGOs with 5, 3, and 2 mm slice thicknesses, obtained at similar doses (1.90–2.13 mGy), are presented in 8, 9, respectively. Though the slice thicknesses and resolution properties were significantly different (2.0–5.0 mm and 0.60–2.11 at 0.50 cycles/mm, respectively), the visibilities of nodules were almost the same, corresponding to their similar SNR_M_^2^ values and visibility scores (HGGO: 5.85–6.98, 3.83–4.22, LGGO: 3.11–4.04, 2.83–3.11, respectively).

**Figure 8:**
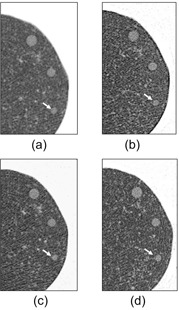
Close‐up images of HGGOs at the middle lung field (white arrows: 6 mm HGGO) for (a) 5 mm slice at 2.10 mGy for image set B, (b) 5 mm slice at 2.13 mGy for image set H, (c) 3 mm slice at 2.07 mGy for image set F, and (d) 2 mm slice at 1.90 mGy for image set D.

**Figure 9:**
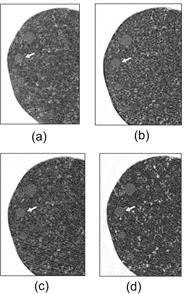
Close‐up images of LGGOs at the middle lung field (white arrows: 10 mm LGGO) for (a) 5 mm slice at 2.10 mGy for image set B, (b) 5 mm slice at 2.13 mGy for image set H, (c) 3 mm slice at 2.07 mGy for image set F, and (d) 2 mm slice at 1.90 mGy for image set D.

## IV. DISCUSSION

SNR_M_^2^ correlated reasonably well with the visual score for both 6 mm HGGOs and 10 mm LGGOs, and is approximately proportional to CTDI_vol_. Accordingly, the visual score is correlated with CTDI_vol_. Furthermore, the rank consistency between the visibility, SNR_M_^2^, and ${\text{CTDI}}_{\text{vol}}$, which is statistically demonstrated, supported these results. SNR_M_^2^ we used in this study involved not only the slice plane SNR^2^ (MTF^2^/NPS), but also the signal spectrum, S, of the slice image of the nodule (spherical object), which depends on SSP and nodule size. The partial volume effect caused by the slice thickness affected the appearances of nodules, and thus influenced the signal spectrum S. Therefore, it appeared that this consideration of S for the SNR measurement contributed to the good correlation of our results. To our knowledge, the SNR investigation taking into account the effect of slice sensitivity profile on the spherical object visibility has not been reported. Boedeker and McNitt‐Gray^(30)^ measured SNR values of CT images obtained by a MDCT system, using a 10 mm diameter numerically simulated spherical object, for different doses and reconstruction kernels. In that study, the assumed SSP was rectangular (not measured SSP), and the image appearance of the spherical object was almost disc‐like with a uniform contrast because the slice thickness used was 2 mm only.

The ${\text{CTDI}}_{\text{vol}}$ of the image sets ranged from 0.85 to 3.3 mGy and, in this dose range, the linear relationship between SNR_M_^2^ and ${\text{CTDI}}_{\text{vol}}$ was approximately obtained under the conditions with different slice thickness and reconstruction kernels. However, it is possible that the linear relationship is impaired due to the additional noise (enhanced electronic noise) caused by the photon starvation at the lower dose.^(31)^ Further investigations are therefore needed to determine how the SNR_M_^2^ correlates with the ${\text{CTDI}}_{\text{vol}}$ in the dose range including the lower dose.

At the onset of this study, we suspected that slice thicknesses of 3 mm or less might not offer sufficiently good visibility because of the increased noise associated with thin slices. However, the thin‐slice image sets obtained in our study did not necessarily offer inferior results in either visibility or SNR_M_^2^. In the image examples with similar doses shown in 8, 9, nodule visibilities of the 2 and 3 mm thickness images (image sets F and D, respectively) are similar to those of the 5 mm thickness images (image sets B and H) because of the lessened partial volume effect due to the thin slice thicknesses. This similarity extends to visibility score (B: 4.22, H: 4.03, F: 3.83, and D: 4.05 for 6 mm HGGOs; B: 3.11, H: 3.02, F: 2.89, and D: 2.83 for 10 mm LGGOs) and SNR_M_^2^ (B: 6.98, H: 6.85, F: 5.85, and D: 5.91 for 6 mm HGGOs; B: 4.04, H: 3.92, F: 3.17, and D: 3.11 for 10 mm LGGOs).

The image sets used in this study had a wide range of MTFs. Among the image sets, the use of enhancement‐type reconstruction kernels did not necessarily improve the nodule visibility, or the enhanced noise of such kernels did not necessarily degrade the nodule visibility. For example, the MTF values of image set B (8, 9) and H (8, 9) at 0.25 and 0.50 cycles/mm were (B) 0.93 and 0.52 and (H) 1.33 and 2.04, and their dose levels were similar, as mentioned above. Though image set H was reconstructed using an enhancement‐type kernel, the nodule visibility of this set is similar to that of image set B, corresponding to their similar visibility score and SNR_M_^2^ values. The ${\text{SNR}}_{M}$ forms a prewhitening type filter that decorrelates the noise, and thus mitigates the effect of the reconstruction kernel. This SNR model is thus considered as a model observer with an ideal detector. Another matched filter type SNR, the non‐prewhitening matched filter SNR (${\text{NPWSNR}}_{M}$) does not decorrelate the image noise.^(32)^ The ${\text{NPWSNR}}_{M}$ is considered as a model observer which measures the noise with respect to how it interacts with the signal, in which the noise enhanced by the enhancement‐type kernel is taken into account. Loo et al.^(23)^ investigated the relationship between visual detectability and eight types of SNR models for analog radiographs of nylon beads with diameters 1.59–3.18 mm. In that paper, the performances of ${\text{SNR}}_{M}$ and ${\text{NPWSNR}}_{M}$ were almost identical, even though the radiographies had different resolution properties. Richard and Siewerdsen^(32)^ reported that the ${\text{SNR}}_{M}$ and ${\text{NPWSNR}}_{M}$ performed similarly in the detection task for a 3 mm diameter sphere on dual energy X‐ray images provided a wide range of image characteristics, since the frequency spectra of the sphere object's signal weighs mostly low spatial frequencies. In our study, the six observers gave similar visibility scores to image set B (roll‐off‐type kernel) and H (enhancement‐type kernel) with the same slice thickness and the similar doses, as demonstrated in the image appearances of 8, 9. In addition, the ${\text{CTDI}}_{\text{vol}}$ was nearly dominant in the GGO visibility through the image sets. Therefore, it seemed that the difference of the resolution property (reconstruction kernel) did not affect the visibility because of the low frequency‐weighted spectra of the 6 mm HGGO and 10 mm LGGO, and the ${\text{SNR}}_{M}$ was consequently effective for evaluating the visibility of synthetic GGOs we used. However, since the number of image sets was limited and the visibility fluctuations between the observers were not small, further investigations are desired to clarify the effect of reconstruction kernel on visibility of spherical objects in CT images.

In image sets with ${\text{CTDI}}_{\text{vol}}\geq 2.0\;\text{mGy}$, the rate of the number of scores ≥3 (3: minimum acceptance level) to total number of score ranged from 94.4% to 100%. Accordingly, the dose levels ≥2.0 mGy appeared to be sufficient for 6 mm HGGO for the MDCT systems we examined. For LGGOs, although the image sets with the highest ${\text{CTDI}}_{\text{vol}}$ (3.3 mGy) indicated the rates of scores ≥3 near 90%, a slight possibility of failure to detect remains. The visibility score value at 2.0 mGy, estimated using the regression line in Fig. 7(a), was 3.91, and thus the estimated ${\text{CTDI}}_{\text{vol}}$ value for 10 mm LGGO, indicated by the same visibility score on the regression line in Fig. 7(b), was 4.2 mGy.

The shape of the synthetic nodules used in this study is assumed to be spherical with uniform content. With this approximation, synthetic nodules compared to actual heterogeneous nodules can potentially overestimate the visibility of the nodule due to higher contrast at the spherical edge.^(12)^ The advantage of using a consistent nodule shape is that it can allow for a more direct comparison of the image sets that would otherwise be not possible using nodules with a complicated shape. In this study, observers made an effort to evaluate the visual score, considering GGO detection tasks in their previous clinical experience in reading lung CT images.

Iterative image reconstruction (IR) techniques have become available in more recent MDCT systems, and can reduce image noise while also preserving resolution. However, the SNR (detectability) evaluation methods for IRs have not been yet standardized, while some methods for physical measurement and detectability evaluation using the model observers have been proposed.^33^, ^34^ According to recent reports, IRs have potentials for reducing the dose levels of LDCT examinations by 35% to 65%, as compared with the filter back projection (FBP), which have been the conventional reconstruction algorithm of CT systems.^35^, ^36^, ^37^ The image sets used in this study were all reconstructed by the FBPs provided in the respective MDCT systems. Thus, the relationship between the GGO visibility and physical detectability obtained in this study does not correspond to the IR images. However, even in the recent MDCT systems available IR, the FBP is implemented and it remains to be the standard reconstruction algorithm. Thus, our results would correspond to the FBP images of the recent MDCT systems, and might contribute to fundamental image quality evaluations for them.

## V. CONCLUSIONS

The visibility of synthetic GGOs in a chest phantom was reasonably correlated with a physical detectability index SNR_M_^2^ that was calculated from measured resolution, noise, and slice thickness properties. Since the SNR_M_^2^ was nearly proportional to the ${\text{CTDI}}_{\text{vol}}$, the GGO visibility was consequently correlated with CTDI_vol_. As a result, the ${\text{CTDI}}_{\text{vol}}$ was nearly dominant in the GGO visibility for the image sets with different reconstruction kernels and slice thicknesses, used in this study.

## ACKNOWLEDGMENTS

This study was supported by a Grant‐in‐Aid for Scientific Research from the Ministry of Health, Labour, and Welfare, and a Grant‐in‐Aid for Scientific Research from the Ministry of Education, Culture, Sports, Science and Technology, Japan. The authors would like to thank Kazuya Funatsu (Freeill Corporation), Kazunari Himematsu (Miyazaki Prefectural Health Foundation), Yuuichi Nakamura (JA Kagoshimaken Kouseiren Health Care Center), Shigemi Ohmachi (Japan Red Cross Nagasaki Genbaku Isahaya Hospital), Ryosuke Mizuno (Nagasaki Municipa Hospital), Hironori Kawakami (Japan Red Cross Kumamoto Health Care Center), Yoshinori Kibe (Ishikawa Health Service Association), Yuji Miyagoshi (Fukuiken Saiseikai Hospital), Yuko Nagano (Niigata Medical Association for Labor Health Plaka Health Care Center), Yuichiro Maruyama (Komoro Kosei General Hospital), Tadahiro Kurata (HOKUSHIN General Hospital), Hiroyuki Fujimoto (Aizawa Hospital), Takahiro Tanikawa (Sekigahara Hospital), and Tsuneo Kobashi (Okayama Kenkoudukuri Zaidan) for their participation in phantom scanning and data recording.
